# Differences in neurocognitive aspects of dyslexia in Dutch and immigrant 6-7- and 8-9-years old children

**DOI:** 10.1186/s40064-015-0874-1

**Published:** 2015-03-01

**Authors:** Johanna MP Verpalen, Fons JR van de Vijver

**Affiliations:** Department of Culture Studies, Tilburg University, P.O. Box 90153, 5000 LE Tilburg, The Netherlands

**Keywords:** Dyslexia, Immigrants, Culture, Neurocognitive basis, Bilinguals, Reading and spelling development

## Abstract

Detecting dyslexia in immigrant children can be jeopardized because of assessment bias, as a consequence of a limited word lexicon or differences in language development of these children. This is in contrast with the view of the universal neurocognitive basis for dyslexia. In this research, differences in screening children at risk for dyslexia with the Dyslexia Screening Test (DST) were studied in third and fifth graders of primary school of Dutch (mainstream) and immigrant descent. Mean group differences were found on a few subtests (Naming Letters, Semantic Fluency, Backward Digit Span and Verbal Fluency), probably as a consequence of bias because of the linguistic character of these subtests. The raw scores of word lexicon increased in the Dutch and immigrant group. The association of having a dyslexia diagnosis on DST scores was comparable for Dutch and immigrant children. Differences in the DST scores between non-dyslexic and dyslexic children were found between the third and fifth grade, with a stronger effect of having a dyslexia diagnosis in the fifth grade than the third grade, for Dutch as well as immigrant children. Screening of dyslexia seems easier in the fifth grade than in the third grade, dyslexic children show a slower reading development than their non-dyslexic peers, irrespective of their cultural background.

**Electronic supplementary material:**

The online version of this article (doi:10.1186/s40064-015-0874-1) contains supplementary material, which is available to authorized users.

## Introduction

Dyslexia is defined as a disorder in reading skills, reading, and spelling development. Dyslexia can affect children’s learning possibilities in a negative way, which makes it very important to detect dyslexia as early as possible in the school career. Early assessment and intervention are important issues in research, as these can enable dyslexic children to live in line with their real potential and intelligence. In the present study we want to detect differences between Dutch and immigrant children in neurocognitive aspects of dyslexia, phonologic awareness, rapid naming, and verbal memory (Goswami 2008), measured with the Dyslexia Screening Test (DST). The purpose of this study is to detect differences in DST scores between 6-7- and 8-9-years old dyslexic and non-dyslexic children, presumably related to the different stages of brain development in reading and spelling area during the development of literacy skills in the two grades. In addition, we set out to identify cultural bias in DST subtests by comparing (dyslexic and non-dyslexic) mainstream Dutch and immigrant children taking into account group differences in level of Word Lexicon.

### Literacy development

**Figure 1:**
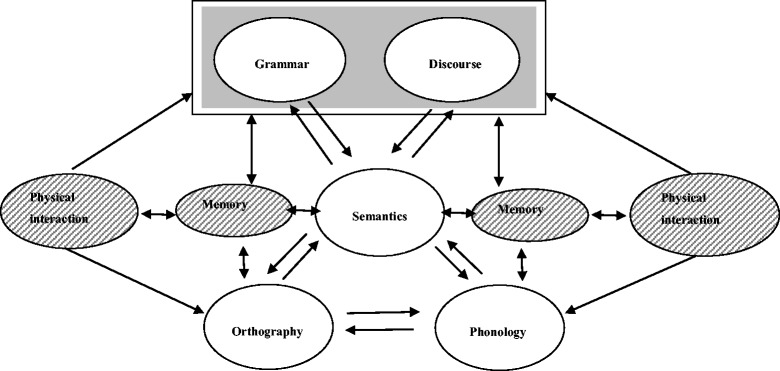
**The triangle framework of reading development (after Seidenberg and McClelland** 1989 **; Bishop and Snowling** 2004 **for the grey part) and recent findings from Glenberg et al. (** 2009 **), Marley et al. (** 2011 **), and Wellsby and Pexman (** 2014 **) added (striped part).**

Oral language, syntax, vocabulary, and phonological processing skills play an important role in early reading development in both first- and second-language learning (Gottardo et al. 2008; Share and Stanovich 1995; Swanson et al. 2008). The triangle framework of reading development and visual word recognition (Seidenberg and McClelland 1989), a widely used theoretical framework of normal development of reading, has guided the development of a variety of connectionist models of reading development (Snowling and Hulme 2007). According to this model, the development of reading skills depends on the interaction between three aspects of words: their sound (phonology), meaning (semantics), and written form (orthography). Two pathways interact when children learn to read. The phonological pathway relates orthography to phonology (a written word can be translated into its spoken form) and the semantic pathway relates orthography to phonology via semantics (a written word produces direct activation of the meaning of the word, which activates pronunciation) (see Figure 1) (Snowling and Hulme 2007).

In the beginning of reading development, the phonological pathway is often used for letter sound mapping, whereas in a later phase, children rely more on the semantic pathway (Plaut et al. 1996). Because children make use of sentence contexts in combination with decoding rules to read new words, Share (1995) and Bishop and Snowling (2004) have expanded the model to incorporate interactions between semantic representations and other sources of linguistic knowledge, such as grammar and discourse level processing (see Figure 1).

The interaction effect in this model explains why vocabulary knowledge in preschool is one of the predictors of later word-level reading skills and word-reading. Children will have fewer difficulties in learning to read the words that are in their speaking vocabulary in their second language (Catts et al. 1999; Elbro et al. 1998; Metsala and Walley 1998). A small vocabulary knowledge can restrict the number of words available for recognition (Nation and Snowling 1998). Dutch third grade children (6–7 years old) have on average a vocabulary knowledge between 4500 and 5200 words. Turkish and Moroccan children achieve this level of vocabulary knowledge when they are nine years old (in the fifth grade of education) (Kuiken and Vermeer 2005; Verhoeven and Vermeer 1991). Ethnic minority children need two years to develop peer-appropriate communicative language and, between five and seven years to fully develop academic language proficiency (Cummins 1984). A small vocabulary can hamper reading comprehension. Such children cannot consistently index or map written words to the objects the words represent, they can fail to derive meaning to the text. Reading becomes, in this case, more an exercise in ‘word calling’ (Glenberg et al. 2004). Glenberg et al. (2004, 2011) showed with a set of experiments that manipulation with toys of the story can enhance young children’s reading performance, as reflected by both their memory for what they have read and their ability to derive text-based inferences. Learning strategies targeted at developing receptive and productive language skills are positively associated with children’s reading achievement (see Elleman et al. 2009 for an overview). Recent embodiment theories are based on principles that cognitive development depends on physical interaction with the environment and physical interaction with objects associated with a symbolic representation (Glenberg and Robertson 2000; Glenberg 2011; Ramus 2003; Wellsby and Pexman 2014). Recent studies show that embodied effects can also be observed in children’s reading comprehension, to make reading comprehension fast and automatic by linking written words to sensorimotor experience, which is called ‘moved by reading’ (Glenberg et al. 2009). These recent findings are added to Figure 1 to complete this figure.

Another significant predictor of reading and spelling proficiency in an alphabetic script is phonological awareness (Bialystok 2006; Ziegler and Goswami 2005). Phonological awareness is defined as the ability to recognize, identify, or manipulate any phonological unit within a word, be it phoneme, rhyme, or syllable (Ziegler and Goswami 2005). In bilinguals, phonological awareness predicts the levels of reading proficiency in each language (Durgunoğlu et al. 1993). Phonological awareness of children with different language backgrounds develops in a similar manner (Chiappe et al. 2002). Research showed that phonological awareness is a skill that is not restricted to the first language and that appears to transfer to one’s second language (Cisero and Royer 1995; Durgunoğlu et al. 1993; Pang 2009; Verhoeven 1994). Young children, who are learning English as a second language, performed less well than native English speakers on tasks measuring phonological awareness in kindergarten, but these differences tend to disappear in the first year of formal schooling, when children are taught sound-letter correspondences (Chiappe et al. 2002). Chiappe et al. (2002) found that the same underlying skills, alphabetic knowledge, spelling, and phonological processing, were strongly related to literacy acquisition in a second language for children with another linguistic background. These findings support the importance of word lexicon and oral language in the development of reading and spelling. Lindsey et al. (2003) showed that the rate of change of bilingual children’s language ability in both languages predict their letter-word identification abilities in both languages.

### Literacy in a second language

In Europe, migration is one of the main factors of bilingualism and language change (Tabouret-Keller 2006). In the Netherlands, 20% of the population has at least one foreign-born parent (9% in a Western country and 11% in a non-Western country) (CBS 2011). In the past, residents of former Dutch colonies migrated to the Netherlands, including immigrants from Indonesia (1950s), Surinam, the Dutch Antilles, and Aruba (1960s). In the same period (1950s), male guest workers were recruited from Southern Europe for factory work in Western European countries and later (1960s) from Turkey and Morocco (Backus 2006). Political and religious refugees from former East Block countries (1970s) and former Yugoslavia (1980s) formed the major source of migration. After family reunification and family formation (after 1980s), a second generation is now well established and a third generation is coming of age (Backus 2006). Present-day migrants are seeking work, better living conditions, and freedom. Most of them come from Turkey, Northern Africa, the former Yugoslavia and various Eastern European countries (such as Poland and Bulgaria), Asia and the US (Tabouret-Keller 2006). Nowadays, about 14% of primary school pupils in the Netherlands are immigrant or have at least one immigrant parent (CBS 2007). The educational achievements of notably non-Western immigrant children are below those of Dutch mainstream children; in addition, relatively few students enter forms of higher education (Backus 2006; CBS 2007). A low level of proficiency in the majority language and sociocultural factors are often related to poor linguistic and scholastic results (Backus 2006; Hamers and Blanc 2000). Bilingual children and young adults generally have weaker receptive vocabulary knowledge in each language than their monolingual peers (Oller et al. 2007; Portocarrero et al. 2007). Most of the immigrant children tend to grow up in a context that is monolingual or dominated by one language, which is the native language of the parents. The mother tongue input decreases when 4-years-old children move into a much more majority language dominated world when they start kindergarten and school (Pfaff 1999). The dominance of the minority language often changes in a majority language dominance after the age of 8 in children of the second and third generation (Akinci et al. 2001; Pfaff 1999). Children of the second and third generation often speak the mother tongue with their parents and the majority language with their siblings, and friends at that age (Backus 2006).

When children learn their first language (mother tongue), they develop a growing knowledge of the world into their continually widening vocabulary, based on their experience and create a system of words and meanings, concepts and symbols, that is core to their intelligence (Bialystok 2001; Smith 2013). Development means learning both concepts to structure the world and words to label and express those structures. Words and concepts do not exist in isolation, but they are organized in networks and are referred to as the “deep structure” of our understanding (Marzano 2004). Bilingual children have different language learning experiences, different cognitive worlds, and are challenged to communicate using different resources (Bialystok 2001). The intellectual path to literacy develops in three stages. The first is the preliteracy stage in which children build up concepts of symbolic representation and learning about the writing system. Bilingual children develop these background concepts differently from monolingual children because of differences in their social, linguistic and cognitive world (Bialystok 2001; Dale et al. 1995). They develop these background concepts for learning to read separately for their two languages, depending on their experience with each (Bialystok 2001).

**Figure 2:**
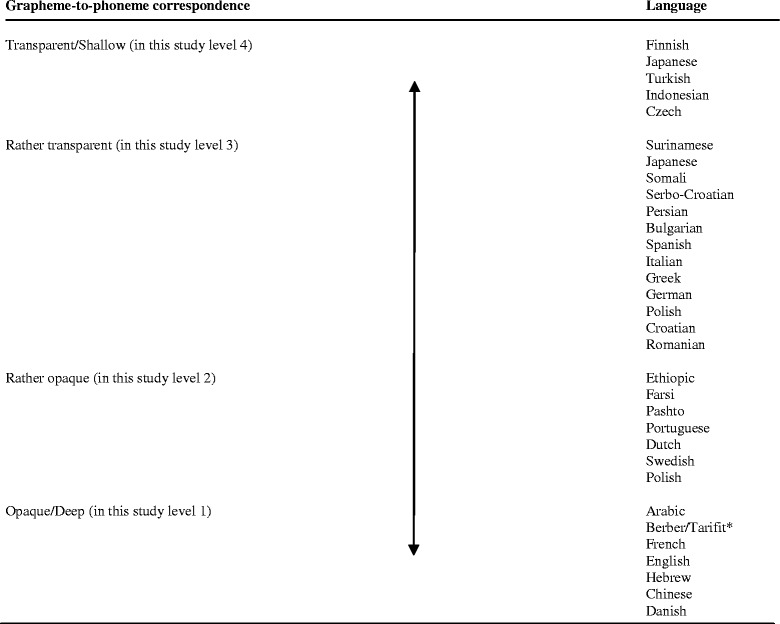
**Level of transparency of diverse languages. Based on: Brunswick (** 2010 **), Seymour et al. (** 2003 **), Smythe et al. (** 2004 **) and Perfetti and Dunlap (** 2008 **).** *Note: Tarifit and Berber were in origin oral languages but are nowadays also written and educated at school since 2002 ( http://www.meertaligheidentaalstoornissenvu.wikispaces.com *)*.

The second is the stage of early learning in which children learn the rules for decoding the written system into the familiar sounds of the spoken language. The first step in an alphabetic script is to learn mapping visual symbols (letters) to units of sounds (phonemes). Differences in reading development are explained by differences in orthography. In some orthographies (e.g., Greek, Italian, Turkish, Spanish and German), letters and letter clusters are almost always spelled and pronounced in the same way (transparent). In other writing systems (e.g., English, Danish and French), letters and letter clusters can have multiple pronunciations and phonemes can have multiple spellings (opaque) (Malloy and Botzakis 2005). The Dutch language is less transparent than Greek and Turkish, but more transparent than English, Danish, and French (Seymour et al. 2003). See Figure 2 for an overview of transparency of diverse languages. The process of learning these mappings is called phonological recoding (Ziegler and Goswami 2005).

Children learn to find shared grain sizes in the symbol system (orthography) and phonology of their language to learn accurate mapping (Goswami et al. 2005; Ziegler and Goswami 2005). Learning to read and spell is easier in a transparent language than in an opaque language (Malloy and Botzakis 2005). A more opaque language like English has a lower mapping consistency at the grapheme-phoneme level, which leads to more variability in the size of grapheme units that need to be combined in the orthography to phonology mappings. Readers in opaque languages like English need to use a larger part (grain size) of the printed word to map onto spoken language, whereas the process of decoding a word letter by letter (small grain size) is more adequate in transparent languages like Turkish (Ziegler and Goswami 2005). In this view, the relationship between vocabulary and reading development should be stronger in less consistent orthographies, where vocabulary can play an important role in recognition of words and parts of words (Ziegler and Goswami 2005). This can be an advantage for bilingual children if their two languages differ in transparency. When children learn the less transparent system, they can profit from this experience in learning the more transparent system of their first language (small grain size strategy) (Bialystok 2001).

The third stage is fluent reading. In this stage, the meaning of the text takes priority and children can begin to use written texts for receiving and expressing ideas, they did not have before (Bialystok 2001). Research showed that the ability in reading fluency in a second language can be predicted by different factors, such as the level of proficiency in the first language (Cummins 1991), the level of proficiency in the second language (Barnett 1989), and the knowledge of cultural schemata and discourse structures of the second language (Barnitz 1986; Carrell 1994; McCardle et al. 2011). Individual differences in reading ability in monolingual and bilingual children are also influenced by reading experience: the more children read, the more skilled they become in reading (Stanovich 1986).

### Dyslexia

Dyslexia has been found in all languages in which it has been studied (for a review, see Smythe et al. 2004). Cross-cultural differences in manifestation are presumably caused by two critical factors: phonological complexity and orthographic transparency of the languages involved (Goswami 2008). There is agreement that children with dyslexia have not developed well-specified phonological representations of the sound structure of the individual words in their mental lexicon (Snowling 2000). These children have difficulties in three kinds of phonological tasks: phonological awareness tasks (e.g., the tapping task and the oddity task), phonological short term memory tasks (digit span) and rapid automatized naming tasks (e.g., naming pictures and naming letters) (Goswami 2008). These difficulties are found in various languages, such as Chinese (Ho et al. 2000), Japanese (Kobayashi et al. 2003), English (Bradley and Byrant 1978), and German (Wimmer 1993) (see Ziegler et al. (2010) for a recent overview).

For children with dyslexia who are learning to read transparent orthographies it is easier to develop the necessary decoding skills than for dyslexic children who are learning to read an opaque language. The impairment in reading speed in dyslexic children means that these children are functionally dyslexic, even if decoding is relatively accurate (Goswami 2008; Ziegler et al. 2010).

### Dyslexia and the brain

Neuroimaging studies show that universal networks for language are left-lateralized to the frontal and temporal areas of the brain of speakers of all languages. For reading, neural networks also seem to be left-lateralized, comprising a network of frontal, temporoparietal, and occipitotemporal regions (Goswami 2008). Learning to read requires associating sounds with letters and the process of automatization of this ability (Haaxma 2006). The starting reader reads by decoding every single letter, a process which takes place in the gyrus angularis and the Broca area. Turkeltaub et al. (2003) found an increase in activity in left temporal and frontal areas in normal reading development, while activity in right posterior areas declined. This pattern shows the possibility that reading-related activity in the brain becomes more left-lateralized with development. Turkeltaub et al. (2003) explored the neural activation associated with phonological awareness. They found that the degree of activity in the left posterior superior temporal cortex and inferior frontal gyrus depends on the level of children’s phonological skills. Analyses of children below 9 years old identified also the left posterior superior temporal cortex suggesting that the route for reading is phonological recoding to sound (Turkeltaub et al. 2003). In this view, it is possible that the 6–7 years old children score different on the DST reading, spelling and phonological tasks than 8–9 years old children.

In fMRI studies, Shaywitz et al. (2002) showed that children with developmental dyslexia showed underactivation in the core brain areas for reading, namely the left frontal, temporal, parietal, and occipital sites, during reading-related tasks (letter identification, single letter rhyme, and nonword rhyming). During these tasks, the right-hemisphere sites, largely in the temporoparietal cortex, were activated by the children with developmental dyslexia (Nicolson and Fawcett 2008; Shaywitz et al. 2002). The role of the cerebellum in automatization was described by Leiner et al. (1989). Recent findings confirmed, in the context of dyslexia, the significance of specific cerebellar activation in reading (Fullbright et al. 1999; see Nicolson and Fawcett 2008 for an overview; Turkeltaub et al. 2002) and working memory (Desmond and Fiez 1998). Nicolson and Fawcett (2008) proposed that cerebellar abnormality from birth leads to slight speech output dysfluency and receptive speech problems (i.e., difficulties in analyzing the speech sounds), and hence to deficiencies in phonological awareness (Nicolson et al. 2001). Taken together with the cerebellar impairment, this analysis could account for the development and pattern of difficulties of dyslexic children.

### Dyslexia in the Netherlands

The age at which children, after a period of kindergarten, begin formal schooling and start with learning to read and spell differs per country. In United Kingdom, formal schooling begins at the age of 5 years (Goulandris 2003), Dutch, Greek, Polish, and American children start at 6 years (Nikolopoulos et al. 2003; Szczerbiński 2003) and children in Germany, Austria, and the Scandinavian countries start when they are 7 years of age (Seymour 2007). Dutch schoolchildren start to learn phonological sensitivity, reading skills and letter-sound correspondence in the first year of education, at the age of four (kindergarten). In the third grade (the first year of formal schooling after two years of kindergarten), they start to learn to read and spell. Every school in the Netherlands has to check the reading and spelling development of its pupils and to identify children at risk for dyslexia.

A well known instrument for identifying children at risk for dyslexia is the Dyslexia Screening Test (DST-NL) (Kort et al. 2005). This instrument was developed in England and translated to Dutch. The target age range is 6.5-16.5 years (Fawcett and Nicolson 2005). The DST (term used for DST-NL in this article) assesses skills that play an important role in dyslexia: literacy skills, rapid naming, working memory, phonological awareness, reading ability and spelling ability. Many subtests of the DST are verbal and have references to the Dutch culture (e.g., Dutch names). These characteristics could affect the immigrant children’s test scores on the DST. When these children start to learn to read in the third grade, they have less experience with the Dutch culture and language. During the years of schooling, vocabulary growth and experience with the Dutch way of education and testing will increase, which will have a positive effect on DST scores. Research shows that the Rapid Naming Pictures, Rapid Naming Letters and Verbal Fluency subtests of the DST are relatively difficult for 8- and 9-years old immigrant children, probably because of the linguistic and cultural character of these subtests (Verpalen and Van de Vijver 2011). Group differences in performance disappeared after statistically controlling for the level of word lexicon (Verpalen and Van de Vijver 2011).

In the Netherlands, the same prevalence of dyslexia has been reported among Dutch (term used here to denote the mainstream group) and immigrant children (Wentink and Verhoeven 2004). Yet, it is difficult to recognize dyslexia in multilingual children and they are under-represented among children assessed as dyslexic (Cline 2000; Peer and Reid 2000). When immigrant children enter schooling, their knowledge of the Dutch language and culture is often limited (Verhoeven 2000). Although differences in language ability between Dutch and immigrant children tend to decrease throughout the school years, they do not disappear (Dagevos and Gijsberts 2007; Voortgangsrapportage 2004). Turkish and Antillean immigrant children are on average still lagging behind two and a half years in language ability at the end of primary school (after eight years of education), Moroccan children two years and Surinamese children one year (Nieuwenhuizen 2005).

Another cause of differences in test scores between Dutch and immigrant children are unintentional difficulties of an instrument, which can have an adverse impact on scores of immigrant children. These factors are referred to as bias (Van de Vijver and Leung 1997). In cross-cultural psychology, three types of bias are distinguished: construct bias, method bias, and item bias (Van de Vijver and Leung 1997). There is construct bias when the test does not measure the same concept across cultures. Method bias refers to measurement anomalies in an instrument arising from particular characteristics of the instrument or its administration, such as tester/interviewer effects, communication problems between respondent and tester, or lack of comparability of samples. Item bias refers to item-specific problems, such as inadequate translation or inadequacy of item content in a cultural group. An item about bacon was more difficult for Islamic children than for Dutch children, because they have less or no contact with it (Resing and Hessels 2001; Van de Vijver and Leung 1997).

### Current study

In this study we aim to detect group differences in DST scores between Dutch and immigrant and between third and fifth graders to address cultural bias in the instrument and to get more insight in the association of development in reading and spelling skills on DST test scores in learning a first and second language. Construct and item bias could challenge the usefulness of the DST, because of the linguistic and cultural character of the DST. Differences in vocabulary knowledge and cultural knowledge between Dutch and immigrant children decrease throughout the school years; so, a decreasing performance gap between immigrant and Dutch children’s DST scores is expected across the period of schooling. This effect is also expected because of the switch in language dominance of minority in majority language after the age of 8 (Akinci et al. 2001; Pfaff 1999), the brain development activity in left temporal and frontal areas in normal reading and the increase in using the semantic pathway in the fluent reading stage, as described in the introduction. The following hypotheses are tested: Differences in DST scores are expected between the non-dyslexic third and fifth graders in both (Dutch and immigrant group) groups and between the dyslexic third and fifth graders in both ethnic groups because of development in literacy skills related to the development in brain activity. Second, lower DST subtest scores and higher dyslexia risk scores are expected in the immigrant group, compared to the Dutch group (construct and item bias could play a role) and these intergroup differences are smaller in the third grade than in the fifth grade, because of the increased level of word lexicon in the fifth grade and the switch in language dominance at the age of 8.

## Method

### Participants

The DST was administered to 125 children in the third year of education (33% Dutch, 67% immigrant) and to 149 children in the fifth year of education (47% Dutch, 53% immigrant). Most of the children of the classes were taking part in this research; a few children did not take part as parents did not give permission for participation. The children were aged 6–7 years (third grade) and 8–9 years (fifth grade), respectively. All participants followed the regular school program of their grade and had sufficient language knowledge for the test administration. In the group of immigrant children, at least one of the parents was foreign born, in most cases in a non-western country. Almost all children were second generation; 44% of the immigrant children were Turkish, 33% were Moroccan, and 23% had other countries of origin, such as Iraq, Vietnam, Indonesia, Surinam and countries in Eastern Europe and Africa. The children were from two different schools with the same teaching methods for education in reading, language and mathematics. Both schools have a relatively high number of dyslexic children, because the schools specialize in dyslexia care in the curriculum. Dutch parents therefore often choose these schools for their children in case of (suspected) dyslexia in their children. A total of 15% were diagnosed with dyslexia (in reading and spelling) of whom 56% were Dutch children and 44% were immigrant children. This means that 21% of the Dutch group and 11% of the immigrant group were dyslexic. The assessment was conducted by psychologists from different centers outside the school using a comprehensive test battery according to the official Dutch dyslexia protocol (Blomert 2006). The test battery measures dyslexia indications (reading ability, spelling ability, phonological awareness, rapid naming, and verbal short term memory).

Many immigrant children speak the (ethnic) mother tongue of the parents at home or a mix of mother tongue (with their parents) and Dutch (with their siblings). The various home languages have different levels of transparency (see Figure 2). In this study, 46.2% of the immigrant children speak a home language with a very high level of transparency (e.g. Turkish, Indonesian, Japanese), 12% a home language with an intermediate level of transparency (e.g. Surinamese, Serbo-Croatian, Somali, Vietnamese), 9% speak a semi-low transparent home language (e.g. Portuguese, Polish, Ethiopic, Farsi, Pashto) and 33% speak an opaque (deep) language at home (e.g. Arabic, Tarifit, Chinese, French, English).

Most of the immigrant participants of this research did not have good Dutch vocabulary knowledge. The level of Dutch vocabulary knowledge (assessed in the same school test on both schools) was divided in five classification groups, based on the standardized scores across grades: very low, low, average, above average and high. In an ANOVA, with culture and grade as fixed factors and Word Lexicon as dependent variable, the effect of culture on Word Lexicon between the Dutch and immigrant third and fifth graders was significant (*F*(3, 268) = 173.57, *p* < .001, ŋ^2^ = .39), the effect of grade was not significant for Word Lexicon (*F*(3, 268) = .005, *p* = .96). As can been seen in Table 1, the Dutch group obtained higher scores. Because of these differences, word lexicon was used as covariate in the analyses, to study the effect of word lexicon on DST scores.

**Table 1:** **Number and percentage of children of the total group per level of Word Lexicon**

**Group**				
**Level of Word Lexicon**	**Dutch**		**Immigrant**	
	**3** ^**rd**^ **graders**	**5** ^**th**^ **graders**	**3** ^**rd**^ **graders**	**5** ^**th**^ **graders**
	**(** ***n*** **= 46)**	**(** ***n =*** **71)**	**(** ***n*** **= 84)**	**(** ***n*** **= 81)**
Very low (1)	1 (2%)	2 (3%)	46 (54%)	35 (44%)
Low (2)	4 (9%)	6 (8%)	16 (19%)	23 (28%)
Average (3)	11 (26%)	18 (25%)	12 (14%)	12 (15%)
Above average (4)	6 (14%)	19 (27%)	5 (6%)	7 (9%)
High (5)	21 (49%)	26 (37%)	6 (7%)	3 (4%)

In the Dutch educational system, the educational level in the home country of the parents is divided in three groups: low (no education or only primary school), middle (primary school and three years low level of high school), and high (at least four years of middle or high school). In this study, 2% of the Dutch and 54% of the immigrant parents had a low educational level, 13% Dutch and 12% immigrant parents had a middle educational level and 85% Dutch and 34% immigrant parents had a high educational level. The difference in the level of education of the parents of the Dutch and immigrant children was significant, χ^2^(2, *N* = 325) = 100.67, *p* < .001). Because of these differences, level of education of the parents was used as covariate in the analyses.

### Measures

The Dutch version of the Dyslexia Screening Test (DST) was administered. The DST has 14 subtests. The DST is a screening test with the purpose to detect children at risk for having dyslexia. After the screening, further research is necessary to diagnose the at-risk children as dyslexic. The interpretation of DST scores is straightforward in that lower scores point to a higher risk of having dyslexia. The risk indicator (called PLQ, PsychoLinguistic Quotient, see Table 2) is based on seven subtests: Rapid Naming Pictures, Rapid Naming Letters, One-Minute Reading, Two-Minutes Spelling, Nonsense Passage Reading, Non-Word Reading and One-Minute Writing. The other subtests are an indication of memory functioning (Phonemic Segmentation 1 and 2, and Backward Digit Span, see Table 2) and Association (Verbal fluency and Semantic fluency, see Table 2). They are not part of the risk indicator of the DST, but still provide indications of dyslexia (Blomert 2006). All subtests were administered, with the exception of Physical Ability (Postural Stability and Bead Threading), because Fawcett and Nicolson (2005) reported no significant relationship between Physical Ability and dyslexia.

**Table 2:** **DST subtests**

**DST factor**	**Subtest names**	**Description**
Psycholinguistic Quotient (PLQ)		
	Rapid naming Pictures	The child has to name correct and as rapidly as possible the name of 50 pictures (5 different objects: chair, tree, duck, knife and bicycle)
	Rapid naming Letters	The child has to name 50 letters as correctly and as rapidly as possible
	One-Minute Reading	The child has to read 24 one syllable words, 24 two syllable words and 24 three syllable words as correctly and quickly as possible
	Two-Minutes Spelling	The child has to spell as many as possible words in two Minutes as correctly and quickly as possible. The number of correctly spelled words in two minutes is the score of the test
	Nonsense Passage Reading	The child has to read aloud, as correctly and quickly as possible a passage that have 10 nonsense words mixed into the sentences of real words
	Non-Word Reading	The child has to read 24 one syllable non-words, 24 two syllable non-words and 24 three non-words as correctly and quickly as possible
	One-Minute Writing	The child has to correctly copy a passage (length is age dependent) as quickly as possible
Memory function		
	Phonemic Segmentation	The child has to segment words into basic sounds and manipulate these words by delete a letter in a word (Segmentation 1) and switch the first letters of the first name and second name of Dutch famous persons (Phonemic Segmentation 2)
	Backward Digit Span	Series of spoken digits are presented to the child. The Child has to repeat the sequence in backward order
Association		
	Verbal Fluency	In one minute, the child has to mention as many as names of words as possible starting with the letter S. The score is the number of correctly mentioned Dutch words
	Semantic Fluency	In one minute, the child has to mention as many as names of animals as possible. The score is the number of animals mentioned

The spelling, reading and vocabulary scores were obtained from school records. Scores on the spelling test (CITO LOVS Spelling), word reading test (CITO LOVS DMT), and word lexicon school test (CITO LOVS Word lexicon) were administered in both schools in January, the middle of the third and fifth year of education. Information about the level of parental education in their mother country was collected from school records. A *Reading School Test (CITO LOVS DMT)* was administered individually, in a separate room during the lessons by an intern. Children have to read as many words as possible in one minute. In the *Spelling school test (CITO LOVS Spelling)* children have to write words, read aloud by the teacher. The test starts with a shared part and is followed by two different parts with different difficulties, depending on the score of the first part. A *Word lexicon school test (CITO LOVS Word lexicon)* measures passive word lexicon, children have to choose the correct meaning of a word from four descriptions of the word.

### Procedure

The DST was administered individually in a quiet room. Three testers were trained in administering the DST. They worked at both schools, two as a remedial teacher and one as a psychologist. The reading, spelling, and word lexicon school tests were administered by the teacher in the class. Data were collected over a period of 6 years (2006–2011).

## Results

### Hypothesis testing

In a MANOVA with culture (Dutch vs. immigrant), diagnosis (non-dyslexic vs. dyslexic), and grade (third vs. fifth grade) as fixed factors and the school test scores for Reading, Spelling and Word Lexicon (divided in five classification groups, based on the standardized scores across grades: the levels 1 (very low) till 5 (high) for every grade separately) as dependent variables, the multivariate effect of grade was significant, Wilks’ Ʌ = .75, (*F*(16, 242) = 5.11, *p* < .001, (partial) ŋ^2^ = .25, which refers to a large effect. We used threshold values for small, medium and large sizes of .01 (small), .06 (medium), and .14 (large) (Cohen 1988). Univariate tests revealed that the observed effect size on the Spelling school test was significant (*F*(1, 257), *p* < .005, ŋ^2^ = .05. The mean score of the third grade (3.70) was larger than the mean score of the fifth grade (3.15) (see Table 3). There was no significant effect of grade on the reading school test scores and word lexicon. There was a multivariate significant effect of the dyslexia diagnosis, Wilks’ Ʌ = .64, *F*(16, 242) = 8.43, *p* < .001, ŋ^2^ = .36. The univariate tests revealed that the observed effect size on the Reading school test scores was large (*F*(1, 257) = 95.84, *p* < .001, ŋ^2^ = .27), with a higher mean score (see Table 3) for the non-dyslexic group (3.75) than the dyslexic group (1.95). The effect size was also large for the Spelling school test scores (*F*(1, 257) = 36.69, *p* < .001, ŋ^2^ = .13), with also a higher mean score (Table 3) for the non-dyslexic group (3.60) compared to the dyslexic group (2.32). There was no significant effect of the dyslexia diagnosis on word lexicon. The multivariate effect of culture was significant, Wilks’ Ʌ = .68, *F*(16, 242) = 7.20, *p* < .001, ŋ^2^ = .32. The univariate tests revealed that (only) the observed effect size on word lexicon was large (*F*(1, 257) = 75.10, *p* < .001, ŋ^2^ = .23). The mean score (Table 3) of the Dutch children (3.90) was higher than the mean score of the immigrant children (1.96). A significant interaction effect was found between grade and diagnosis, Wilks’ Ʌ = .90, *F*(16, 242) = 1.78, *p* < .05, ŋ^2^ = .11. The univariate tests revealed that the observed effect size was significant, yet small (*F*(1, 257) = 5.06, *p* < .05, ŋ^2^ = .02) for Phonemic Segmentation 1 and also small (*F*(1, 257) = 4.29, *p* < .05, ŋ^2^ = .02) for Non-Word Reading. The mean scores (see Table 3) on Phonemic Segmentation 1 of the non-dyslexic children (9.35) and the dyslexic children (8.89) in the third grade were lower than the mean scores of the non-dyslexic children (9.88) and dyslexic children (7.86) in the fifth grade. The non-dyslexic third graders (9.20) scored lower than the non-dyslexic fifth graders (10.00) on Non-Word reading, and the dyslexic third graders (6.22) scored higher than the dyslexic fifth graders (5.33) on Non-Word Reading.

**Table 3:** **Standardized mean scores Dutch non-dyslexic and dyslexic third and fifth graders and immigrant non dyslexic and dyslexic third and fifth graders**

	***Dutch***		***Dutch***		***Immigrant***		***Immigrant***	
	***3*** ^***rd***^ ***graders***		***5*** ^***th***^ ***graders***		***3*** ^***rd***^ ***graders***		***5*** ^***th***^ ***graders***	
	***Non-dyslexic***	***Dyslexic***	***Non-dyslexic***	***Dyslexic***	***Non-dyslexic***	***Dyslexic***	***Non-dyslexic***	***Dyslexic***
Naming Pictures	10.28	9.33	9.70	8.71	9.28	8.56	9.57	7.78
Naming Letters	11.56	9.22	11.68	9.93	11.15	9.00	9.53	8.67
One-Min. Reading	9.19	4.67	10.84	6.29	8.20	4.78	9.66	6.67
Phon. Segment. 1	10.00	8.78	10.63	8.64	9.29	8.78	9.56	8.33
Phon. Segment. 2	9.91	8.78	10.54	7.71	9.08	9.00	9.33	7.78
Two-Min. Spelling	10.63	8.11	10.16	7.07	9.63	8.78	10.01	8.56
Backw. Digit Span	9.50	8.11	9.66	9.36	8.79	9.00	9.70	11.22
Nons. Pass. Reading	10.22	7.89	10.71	8.07	9.79	8.33	10.49	7.56
Non-Word Reading	9.50	5.67	9.48	5.21	8.95	6.78	10.26	5.33
One-Minute Writing	11.19	9.00	10.11	7.29	10.35	8.89	10.27	8.67
Verbal Fluency	10.97	7.89	11.00	9.21	10.40	11.00	10.01	11.11
Semantic Fluency	10.84	10.33	10.64	9.71	9.09	9.67	9.09	9.11
PLQ	103.25	83.89	102.63	82.14	97.64	85.22	99.71	83.56
Reading school test	3.69	2.22	4.16	2.00	3.55	1.56	3.83	2.00
Spelling school test	3.94	2.67	3.53	1.62	3.78	3.22	3.29	2.11
Word Lexicon school test	3.91	4.11	3.98	3.46	1.95	1.89	1.91	2.56

The other interaction effects were not significant (between grade and culture: Wilks’ Ʌ = .94, *F*(16, 242) = .94, *p =* .53, ŋ^2^ = .06; between culture and diagnosis: Wilks’ Ʌ = .92, *F*(16, 242) = 1.27, *p* = .22, ŋ^2^ = .08; between grade, culture and diagnosis: Wilks’ Ʌ = .95, *F*(16, 242) = .87, *p* = .61, ŋ^2^ = .05. In sum, we found significant effects of grade (on the Spelling school test), dyslexia diagnosis (on the Reading school test and Spelling school test) and culture (on Word Lexicon) and an interaction effect between grade and dyslexia diagnosis on Phonemic Segmentation 1 and Non-Word Reading.

It could be argued that the previous MANOVA did not consider covariates that could potentially account for existing cultural differences. We addressed the influence of two relevant confounding variables in the next analysis: the level of parental education and word lexicon. We conducted a MANCOVA, with group (third vs. fifth graders), and culture (Dutch vs. immigrant), diagnosis (non-dyslexic vs. dyslexic), and grade (tested in the third or fifth grade) as fixed factor and the standardized scores on each DST subtest and PLQ as dependent variables and the effect of parental education and word lexicon as covariates. This analysis tested to what extent word lexicon and parental education could explain the cross-cultural differences in DST scores. In the MANCOVA, the multivariate effect of word lexicon (Wilks’ Ʌ = .87, *F*(13, 250) = 2.99, *p* < .001, ŋ^2^ = .14), grade (Wilks’ Ʌ = .80, *F* (13, 250) = 4.71, *p* < .001, ŋ^2^ = .20), culture (Wilks’ Ʌ = .90, *F*(13, 250) = 2.08, *p* < .05, ŋ^2^ = .10) and diagnosis (Wilks’ Ʌ = .74, *F*(13, 250) = 6.91, *p* < .001, ŋ^2^ = .26) were significant. However, the multivariate effect of parental education was not significant, Wilks’ Ʌ = .95, *F*(13, 250) = 1.08, *p* = .38, ŋ^2^ = .05. The multivariate effect of the interaction between grade and having a diagnosis was significant, Wilks’ Ʌ = .91, *F*(13, 250) = 1.83, *p* < .05, ŋ^2^ = .09, which refers to a medium effect size. The multivariate interaction between grade and culture was not significant, Wilks’ Ʌ = .96, *F*(13, 250) = .90, *p* = .55, ŋ^2^ = .05, and the effect of interaction between culture and having a diagnosis was not significant, Wilks’ Ʌ = .95, *F*(13, 250) = 1.06, *p* = .39, ŋ^2^ = .05. The effect of the interaction between grade, culture and having a diagnosis was not significant, Wilks’ Ʌ = .97, *F*(13, 250) = .64, *p* = .82, ŋ^2^ = .03.

A significant, yet small effect of culture was found on Naming letters (see Table 4) before controlling for word lexicon and parental education. There was also a significant effect of culture found on Semantic Fluency, which was small. After controlling for word lexicon and the level of education of the parents, there was a significant, small effect of culture on Backward Digit Span and Verbal Fluency. After controlling for word lexicon and level of education of the parents, the effect of culture was no longer significant for the subtests Naming Letters and Semantic Fluency. The effect of grade was significant for One-Minute Reading and Backward Digit Span. The effect of having a dyslexia diagnosis was significant for Naming Letters, Naming Pictures, One-Minute Reading, Phonemic Segmentation 1, Phonemic Segmentation 2, Two-Minutes Spelling, Nonsense Passage Reading, Non-Word Reading, One Minute Reading and for the PLQ. The effect of grade on One-Minute Reading and Backward Digit Span was still significant after controlling for word lexicon and the level of education of the parents.

**Table 4:** **Multivariate analysis of variance of culture, grade and diagnosis, before and after correcting for the effect of Word Lexicon and educational level of the parents as covariates (total group)**

	***Culture***		***Grade***		***Diagnosis***	
	***Correction***		***Correction***		***Correction***	
***DST subtest***	***Before***	***After***	***Before***	***After***	***Before***	***After***
Naming Pictures	.01	.01	.00	.00	.02*	.01
Naming letters	.02*	.00	.00	.00	.05***	.05***
One-Min. Reading	.00	.00	.04***	.04***	.18***	.18***
Phon. Segment. 1	.01	.00	.00	.00	.03**	.03**
Phon. Segment. 2	.01	.00	.00	.00	.06***	.06***
Two-Min. Spelling	.00	.01	.00	.00	.10***	.09***
Backw. Digit Span	.00	.03**	.02*	.01*	.00	.00
Nons. Pass. Reading	.00	.00	.00	.00	.13***	.12***
Non-Word Reading	.00	.00	.00	.00	.27***	.24***
One-Minute Writing	.00	.00	.01	.01	.08***	.07***
Verbal Fluency	.01	.02**	.00	.00	.01	.01
Semantic Fluency	.03**	.00	.00	.00	.00	.00
PLQ	.00	.00	.00	.00	.18***	.16***

*p<.05. **p<.01. ***p<.001.

As can been seen in Table 4, the effect size of having a dyslexia diagnosis is between small and medium for the subtests Naming Pictures, Naming Letters and Phonemic Segmentation 1, medium for the subtest Phonemic Segmentation 2, between medium and large for the subtest Two-Minutes Spelling and Nonsense Passage Reading, and large for One-Minute Reading, Non-Word Reading and the PLQ (Table 4). In all cases dyslexia was associated with lower performance. The effect of having a dyslexia diagnosis on the DST subtests showed a similar pattern after controlling for word lexicon and educational level of the parents, the effect of having a dyslexia diagnosis had a significant effect on the same subtests with a comparable weight (see Table 4), with the exception Naming Pictures, the effect of a dyslexia diagnosis was no longer significant in the MANCOVA, although the reduction in effect size was very modest. Differences in mean scores between the third and fifth grade were found on One-Minute Reading (effect between small and medium) and Backward Digit Span (small effect).

In a third analysis, involving only the immigrant group a MANCOVA was conducted to evaluate the effect of the level of transparency in the home language. In the MANCOVA with diagnosis (non-dyslexic vs. dyslexic), and grade (third vs. fifth grade) as fixed factor and the standardized scores on each DST subtest and the PLQ as dependent variables and the effect of parental education and word lexicon as covariates, the level of transparency was added as covariate too. The multivariate effect of the level of transparency of the home language was not significant, Wilks’ Ʌ = .93, *F* (13, 153) = .88, *p* = .57, η^2^ = .07. There was only a significant effect, between small and medium, of transparency of the home language found on the subtest Naming Letters (*F* (3, 168) = 4.83, *p* = .03, η^2^ = .03).

Because of the significant interaction effect between grade and having a diagnosis, mean differences in the DST scores between the non-dyslexic and dyslexic children in the third and fifth grade separately, were further analyzed in a set of *t* tests. In the third group, the non-dyslexics scored significantly higher on seven subtests and the PLQ (Table 5), with a small effect size on Phonemic Segmentation 2, a medium effect size on Two-Minutes Spelling and One-Minute Writing and a large effect size on Naming Letters, One-Minute Word Reading, Nonsense Passage Reading, Non-Word Reading and the PLQ compared to their dyslexic classmates (absolute values Cohen’s *d* for small, medium and large sizes: 0.2 (small), 0.5 (medium) and 0.8 (large); Cohen, 1988). In the fifth grade, the non-dyslexic children scored significant higher on eight subtests and the PLQ, with a small effect size on Naming Pictures, a medium effect size on Phonemic Segmentation 1, and a large effect size on One Minute Reading, Phonemic Segmentation 2, Two Minute Spelling, Nonsense Passage Reading, Non-Word Reading, One Minute Writing and the PLQ (see Table 5), compared to their dyslexic classmates. As can be seen in the table, the large effect of a dyslexia diagnosis on Naming Letters in the third grade is no longer significant in the fifth grade. In contrast to the third grade there was a significant small effect of a dyslexia diagnosis on Naming Pictures and a medium effect on Phonemic Segmentation 1 in the fifth grade. An increase of effect sizes in the fifth grade was found in One-Minute Word Reading, Phonemic Segmentation 2, Two-Minutes Spelling, Nonsense Passage Reading, Non-Word Reading, One-Minute Writing and the PLQ (Table 5). In sum, the differences in DST scores and the PLQ increased between the third and fifth grade.

**Table 5:** **Differences in mean scores DST subtests between non-dyslexic and dyslexic third and fifth graders**

	**Third grade**			**Fifth grade**		
**Subtest**	**Non-dyslexic**	**Dyslexic**	***d***	**Non-dyslexic**	**Dyslexic**	***d***
Naming Pictures	9.58	8.94	.22	9.63	8.35	.41*
Naming Letters	11.27	9.11	.76**	10.48	9.43	.36
One-Min. Reading	8.50	4.72	1.39***	10.18	6.43	1.68***
Phon. Segmentation 1	9.50	8.78	.29	10.03	8.52	.61*
Phon. Segmentation 2	9.33	8.89	.35*	9.87	7.74	.90***
Two-Minutes Spelling	9.93	8.44	.71**	10.08	7.65	1.38***
Backward Digit Span	9.00	9.06	-.02	9.68	10.09	-.14
Nons. Passage Reading	9.92	8.11	.95**	10.59	7.87	1.19***
Non-Word Reading	9.11	6.22	1.30***	9.91	5.26	2.20***
One-Minute Writing	10.60	8.94	.63*	10.20	7.83	.89***
Verbal Fluency	10.57	9.44	.31	10.45	9.96	.15
Semantic Fluency	9.62	10.00	-.14	9.78	9.48	.13
PLQ	99.32	84.56	1.16***	101.10	82.70	1.48***

*p<.05, **p<.01, ***p<.001.

In summary, differences in DST scores between the non-dyslexic and dyslexic third and fifth graders (Dutch and immigrant) (hypothesis 1) were found in both groups. We found a significant effect of having a dyslexia diagnosis in the Dutch and immigrant group. Noticeable is the interaction between diagnosis and grade. The score differences between the dyslexic and reference group increased with grade. Contrary to our expectation, a significant effect of culture on DST subtests scores and the PLQ was only found on a few subtests and the effects were small: Naming Pictures and Semantic Fluency before controlling for Word Lexicon and Educational Level of the parents and Backward Digit Span and verbal Fluency after controlling for Word Lexicon and Educational level of the parents (hypothesis 2). There was no significant interaction between culture and grade, we did not find a decrease of cultural differences from the third to the fifth grade (hypothesis 2).

## Discussion

The purpose of this study was to detect differences in neurocognitive aspects of dyslexia in Dutch and immigrant 6-7- and 8-9-years old children, with the expectation that more differences would be found in the third grade (6-7-years old children) than in the fifth grade (8-9-years old children). These expectations were based on the presumed development of reading and spelling skills between the third and fifth grade, the language development of the immigrant children during the period of schooling between the third and fifth grade, development of brain activity during reading and spelling tasks, bias in the screening test or dyslexia assessment and other assessment difficulties. However, in this research, we found the opposite; the differences between non-dyslexic and dyslexic children increased with grade, the lag in development increased in both dyslexic Dutch and immigrant children. When dyslexia is diagnosed, the difficulties develop in a comparable way for Dutch and immigrant children, which is in line with the notion that phonological awareness, phonological short term memory, and rapid automatized naming are universal predictors of dyslexia (Chiappe et al. 2002; Goswami 2008; Snowling 2000; Ziegler et al. 2010), which are represented in the DST. These data are compatible with the view that dyslexia is a neurodevelopmental disorder with a salient genetic component. Paulesu et al. (2001) showed a universal neurocognitive basis for dyslexia across languages. The aim of their study was to contrast dyslexic and normal readers in deep (English and French) and shallow (transparent) (Italian) orthographies in order to explore similarities and differences at the cognitive and brain level, or both. At the cognitive level, the usual pattern was found: the Italian, English, and French dyslexic group performed more poorly on the phonological short-term memory (digit span, digit symbol), phonological tasks and reading tasks, compared to the control non-dyslexic group. The Italian group differed strongly from their control group (Italian non-dyslexics) on nonword reading, but performed better on the nonword reading task when compared to the French and English dyslexic groups. The phonological impairment in dyslexics supports the idea that dyslexia is associated with a phonological deficit that is independent of orthography (Paulesu et al. 2001), which is confirmed in this study. At brain level, reduced activation has been found in the left middle, inferior and superior temporal cortex and in the middle occipital gyrus in all three language groups. These results suggest that dyslexia has a universal basis in the brain and can, independent of the orthography, be characterized by the same neurocognitive deficit (Paulesu et al. 2001). It seems that the expected differences (differences in phonological tasks and literacy skills between Dutch and immigrant third and fifth graders) were not found because of the universal basis of brain activity and that the Dutch language, reading, and spelling skills, develop in the same way when immigrant children start schooling in the Netherlands from the first grade.

The effect of culture on DST scores was limited. Small differences in the effect of culture before and after controlling for word lexicon and parental education were found in the subtests Naming Letters, Backward Digit Span and Verbal and Semantic Fluency. These subtests have a very linguistic character, which could be associated with bias. Further research could make this clear. Probably, the development of reading, spelling and the phonological skills develop in the same way in Dutch and immigrant children, during the schooling period from the first to the fifth grade. The technical character of these skills, which are taught in the educational program from the start, could be an explanation why training effects are similar for both groups. When immigrant children start their Dutch education in the first grade, they follow the same training of these skills as the Dutch children, with probably the same effects. Further research could make clear if there are differences between children with Dutch schooling from the first grade and children who started later with Dutch education, when they arrived in the Netherlands at an older age. Still, the level of word lexicon has a correlation with several subtests: in the third grade on the subtests Phonemic Segmentation 1 and 2 and Backward Digit Span and in the fifth grade on the subtests One-Minute Reading, Phonemic Segmentation 1 and 2, Backward Digit Span, Verbal Fluency and Semantic Fluency and could have an important influence on the DST scores from immigrant children, in view of their lower scores on the Word Lexicon school test. Recent reading models, with an influence of sensorimotor experience on language development (see Figure 2), especially on semantic knowledge, could also play a role in the development and ability of language skills and, related to this, reading comprehension and reading fluency, semantic fluency and naming fluency. Further research could explore this link.

In this research, the immigrant children scored lower on the word lexicon school test (in both, raw scores and standardized scores across grades as a classification of the level of Word Lexicon) than the Dutch children in the third grade. We expected higher raw Word Lexicon scores in the fifth grade, in such an extent that they reach a higher level of word lexicon classification. However, the raw scores increased in both groups, Dutch and immigrant, but not enough for the immigrant group to reach a higher level of word lexicon in standardized scores. The differences in standardized Word Lexicon scores between the third and fifth grade was nil, in both (Dutch and immigrant) groups, both groups stayed in the same classification of standardized level of word lexicon, the differences between the Dutch and immigrant group (see Table 1) did not disappear in two years of education (between third and fifth grade). The immigrants did not make up for their backlog in word lexicon, in contrast with our expectation and, because of this, the immigrant fifth graders could not profit in DST scores from a higher word lexicon level, which could be an explanation of the limited differences between immigrant third and fifth graders.

Differences between the third (total group) and fifth grade (total group) of this research were found for the dyslexic children. Probably, the DST has a better screening effect in the fifth grade, because there are more significant dyslexia indications with a larger effect found in the fifth grade. Screening of dyslexia seems easier in the fifth grade, differences are clearer, maybe because differences in phonemic awareness, verbal memory and rapid naming are clearer. Dyslexic children show a slower development than their non-dyslexic peers. Further research can show the effect of screening for dyslexia with the DST in a higher grade, for example the seventh or eighth grade.

A limitation of this research was the small number of dyslexic children and the small number of immigrant children with a high level of word lexicon. This is an issue which requires further research. A larger group of immigrant children makes it possible to examine the influence of different mother tongue like Tarifit (language spoken by Rif Berbers, the mother tongue of the Moroccan children in this study) and Turkish.
